# Comparative analysis of microglia-targeted AAVs reveals capsid choice drives efficiency *in vitro* but has limited impact *in vivo*

**DOI:** 10.64898/2026.08.05.739854

**Published:** 2026-08-05

**Authors:** Saša Jereb, Lia R. D’Alessandro, Nader Morshed, Margery Chen, Rafaela Sartore, Zengpeng Han, Jordan E. McKinney, Pamela P. Brauer, John W. Harvey, Matthew Demers, Dara Cuffe, Raphael Rakosi-Schmidt, Luira Leite, Youtong Huang, Qingxia Zheng, Chin-Yen Lin, Ken Y. Chan, Bryan J. Song, Michael R. Farzan, Paola Arlotta, Morgan Sheng, Mariko L. Bennett, Matthew B. Johnson, Beth Stevens, Benjamin E. Deverman

## Abstract

Microglia play key roles in brain development, homeostasis, and neurodegeneration. Although multiple strategies for viral gene delivery to microglia have been reported, they have not been directly compared. Here, we developed microglia-targeting AAV capsids and benchmarked them against existing approaches. The novel capsids exhibit improved transduction efficiency in cultured mouse and human microglia, as well as neurons and astrocytes. However, when we compared microglial transduction efficiency of the novel capsids with published engineered and naturally occurring capsids after intracranial injection, all capsids achieved efficient and specific transduction when paired with a genome incorporating *IBA1* promoter and miR-124 target sites. In contrast, CAG promoter did not support efficient microglial transduction. Moreover, blood-brain barrier- crossing capsids carrying *IBA1* promoter and miR-124 target sites efficiently transduced microglia at high doses but exhibited off-target expression. Together, our work provides improved capsids for *in vitro* manipulation of microglia and establishes viral genome design, not capsid identity, as the principal determinant of efficient *in vivo* microglial targeting.

## Full Text Availability

The license terms selected by the author(s) for this preprint version do not permit archiving in PMC. The full text is available from the preprint server.

